# Identification of the ageing‐related prognostic gene signature, and the associated regulation axis in skin cutaneous melanoma

**DOI:** 10.1038/s41598-022-22259-0

**Published:** 2023-01-11

**Authors:** Chonglin Tian, Sujing Liu, Ran Huo

**Affiliations:** 1 Department of Burn and Plastic Surgery, Shandong Provincial Hospital Affiliated to Shandong First Medical University, Jinan, 250021 Shandong China; 2 Department of Burn and Plastic Surgery, Shandong Provincial Hospital, Cheeloo College of Medicine, Shandong University, Jinan, 250021 Shandong China; 3 Shandong Provincial Third Hospital, Shandong University, Jinan, 250031 Shandong China

**Keywords:** Melanoma, Cancer genomics, Cancer therapy, Tumour biomarkers, Tumour immunology

## Abstract

Skin cutaneous melanoma (SKCM) has substantial malignancy and a poor prognosis. The function of ageing-related genes (ARGs) in SKCM is unknown. In this study, a prognostic risk-scoring model for ARG was constructed based on SKCM RNA-seq, mutation, and clinical data in The Cancer Genome Atlas. Our novel prognostic model, which included four ARGs (*IRS2*, *PDGFRA*, *TFAP2A*, and *SOD2*), could distinguish between low-risk and high-risk groups. Low-risk patients benefited more from immunotherapy and commonly used targeted and chemotherapy drugs than high-risk patients. There were also considerable differences in immunocyte infiltration and tumour microenvironment between the two groups. Furthermore, multivariate Cox regression analysis revealed that age, pT_stage, pM_stage, body mass index, tumour mutation burden, and risk score were independent factors influencing the prognosis of patients with SKCM; therefore, we devised a prognosis nomogram. Last, a long non-coding (lncRNA) *NEAT1*/miR-33a-5p/*IRS2* regulatory axis of the competing endogenous RNA network was built to investigate the mechanisms of SKCM metastasis progression. Grouping based on the scoring system could predict the prognosis of SKCM and predict the sensitivity of patients to immunotherapy, targeted therapy, and chemotherapy. This could facilitate the formulation of individualised treatment strategies and help drug research and development. These findings highlight the regulatory axis of the lncRNA *NEAT1*/miR-33a-5p/*IRS2*, which may play a role in SKCM metastasis.

## Introduction

Skin cutaneous melanoma (SKCM) is a potentially fatal malignancy with an increasing incidence^1^. Although most patients are diagnosed early and can be treated by surgical resection of the primary tumour, numerous patients develop distant metastases, leading to the vast majority of deaths among patients with SKCM. More than half of all SKCM patients were older in 2020, according to worldwide age-standardised incidence estimates; the proportion of patients aged 60 years was 17.8% per 100,000, and the proportion of patients aged 70 years was 26.2% per 100,000^2^. Given the increasing growth and ageing of the global population, the number of older patients with SKCM will continue to increase markedly.

Ageing has been a focus in tumour research^3–5^. Like other ageing-related disorders, tumours become more common in middle and later ages. Cell ageing is a complicated and important factor in tumour development, as it can both inhibit and promote tumour growth^6–8^. For example, H3K9me-mediated cell ageing is a novel mechanism for inhibiting lymphoma formation^9^, whereas cell ageing could limit the occurrence of early prostate cancer tumours^10^. According to the senescence-associated secretory phenotype^11^, several investigations have demonstrated that injecting ageing fibroblasts into immunocompromised mice can greatly increase the proliferation of epithelial tumour cells^12, 13^. Therefore, ageing-related genes (ARGs), which regulate cell ageing, play a key role in tumour occurrence^8, 14^.

The significance of the ageing process in SKCM is uncertain, and the relationship between ARGs and SKCM prognosis has not been thoroughly investigated. Therefore, we performed bioinformatic analysis to study the ARG expression profile and its prognostic importance in SKCM, as well as the corresponding regulatory axes, to identify prognostic biomarkers and therapeutic targets for the disease.

## Results

### Expression and mutation of ARGs in SKCM

**Figure 1:**
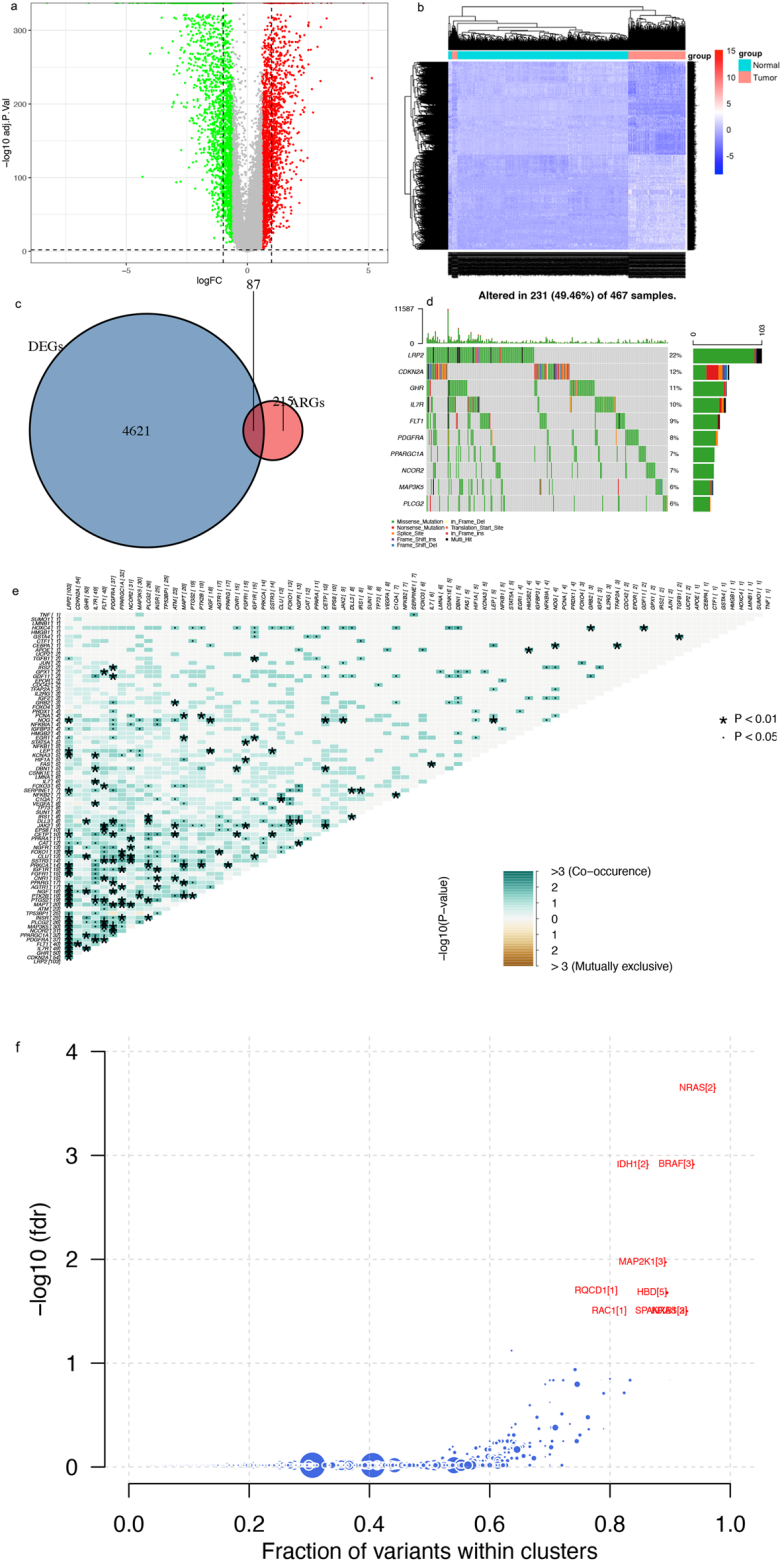
Expression and genetic profiles of ARGs in SKCM. (**a**,**b**) Volcano and heat maps of DEGs in tumour and normal tissues. (**c**) Intersection of DEGs and ARGs. (**d**) Mutation frequency of differentially expressed ARGs. (**e**) Prediction of driver genes. (**f**) Synergy and mutual exclusivity of ARG mutations. *DEG* differentially expressed gene, *ARG* ageing-related gene.

We identified 4708 differentially expressed genes between SKCM in The Cancer Genome Atlas (TCGA) and normal skin tissues in GTEx and took their intersection with 307 ARGs, yielding 87 ARGs, among which 38 were up-regulated and 49 were down-regulated in SKCM (Fig. 1a‒c, Table S1). *GRB2, IRS2, IRS1, JAK2, IGF1R, IL2RG, SUMO1, INSR,* and *PPARGC1A* were identified as hub genes (Fig. S1).

We then summarised the incidence of somatic mutations for the 87 ARGs in SKCM (Fig. 1d). Among the top 10 genes with the highest mutation frequency, 231 of 467 (49.46%) SKCM samples showed gene mutations, primarily missense mutations. *LRP2*, followed by *CDKN2A* and *GHR*, was the most frequent mutant gene. Interestingly, most of the 87 mutated ARGs showed a synergistic effect, but almost none showed a mutual exclusion effect (Fig. 1e).

We also predicted genes that drive the development of malignant melanoma and identified *NRAS, BRAF, IDH1, MAP2K1, RQCD1, HBD, KRAS, RAC1,* and *SPANXB1* as potential driver genes (Fig. 1f).

### Establishment and validation of an ARG-based prognostic model

**Figure 2:**
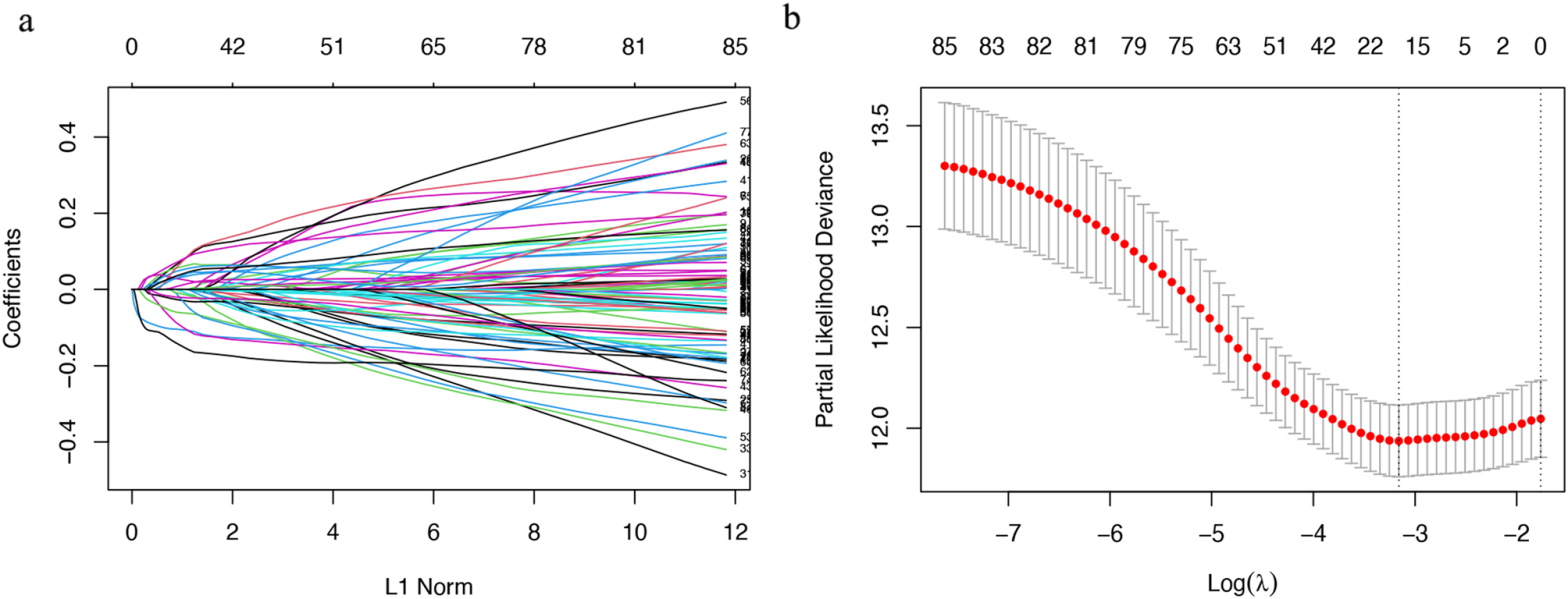
LASSO Cox regression analysis.

To construct a prognostic gene model, by screening 87 candidate ARGs for variables while fitting a generalised linear prognostic model, 18 ARGs with prognostic value were screened by LASSO regression analysis (Fig. 2, Table S2). We identified four genes with an independent prognostic value, namely *IRS2*, *PDGFRA*, *TFAP2A,* and *SOD2*, by Cox multivariate regression analysis (Table S2). A prognostic risk-scoring model was established based on these four prognostic ARGs: risk score = (− 0.2526) × *IRS2* + (0.2585) × *PDGFRA* + (0.1559) × *TFAP2A* + (− 0.2489) × *SOD2*.

**Figure 3:**
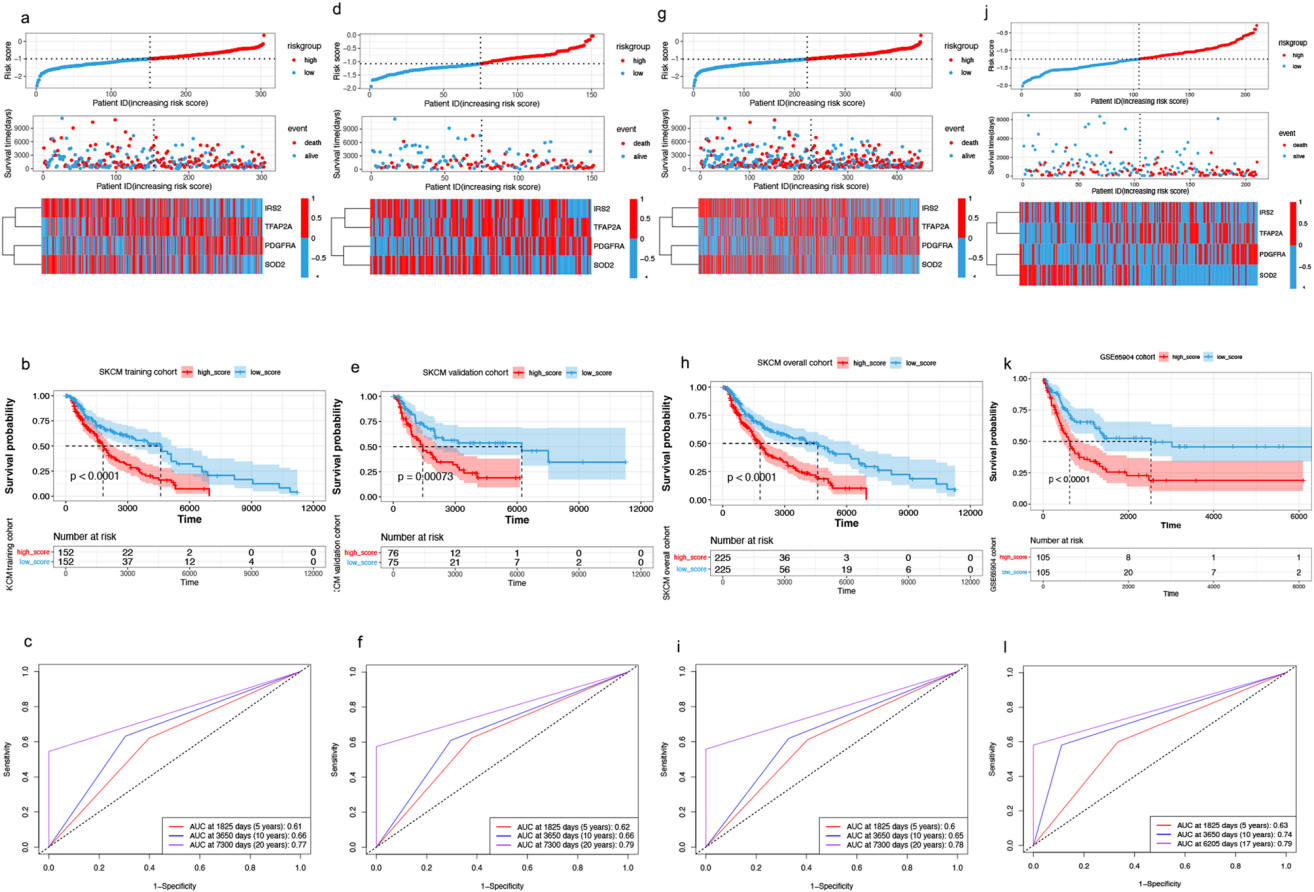
Construction and validation of risk-scoring models. (**a**) Distribution of the risk score, survival status, and expression of four prognostic ARGs in the training queue. (**b**,**c**) Overall survival curves for patients in the high- and low-risk groups and AUCs in the training queue. (**d–f**) Presentation of the above metrics in the validation queue. (**g–i**) Presentation of the above metrics in the overall queue. (**j**–**l**) Presentation of the above metrics in the GSE65904. *ARG* age-related gene, *AUC* area under the receiver operating characteristic curve.

Based on the median risk score, patients with SKCM were divided into two groups. Figure 3a indicates the distribution of the risk score, the survival status, and the expression of ARG in the training set. As the risk score increased, the mortality risk increased, and the overall survival (OS) decreased.

The Kaplan‒Meier curves revealed that patients with a high-risk score had a OS significantly shorter than those with a low-risk score (median time of 1010 days vs. 1429 days, *p* < 0.0001, Fig. 3b), and the areas under the ROC curves (AUCs) at 5, 10, and 20 years were 0.61, 0.66, and 0.77, respectively (Fig. 3c). In the internal validation set, OS was considerably shorter in patients with a high-risk score than in those with low-risk score (median time of 821 days vs. 1490 days, *p* = 0.0007), with AUCs of 0.62, 0.66, and 0.79 at 5, 10, and 20 years, respectively (Fig. 3d‒f). In the total cohort, OS was significantly shorter in patients with a high-risk score than in those with low-risk score (median time of 938 days vs. 1446 days, *p* < 0.0001), with AUCs of 0.60, 0.65, and 0.78 at 5, 10, and 20 years, respectively (Fig. 3g‒i). In the external GSE65904 validation set, OS was significantly shorter in patients with a high-risk score than in those with low-risk score (median time of 455 vs. 638 days, *p* < 0.0001), with AUCs of 0.63, 0.74, and 0.79 at 5, 10, and 17 years, respectively (Fig. 3j‒l).

### Establishment of predictive nomograms

**Figure 4:**
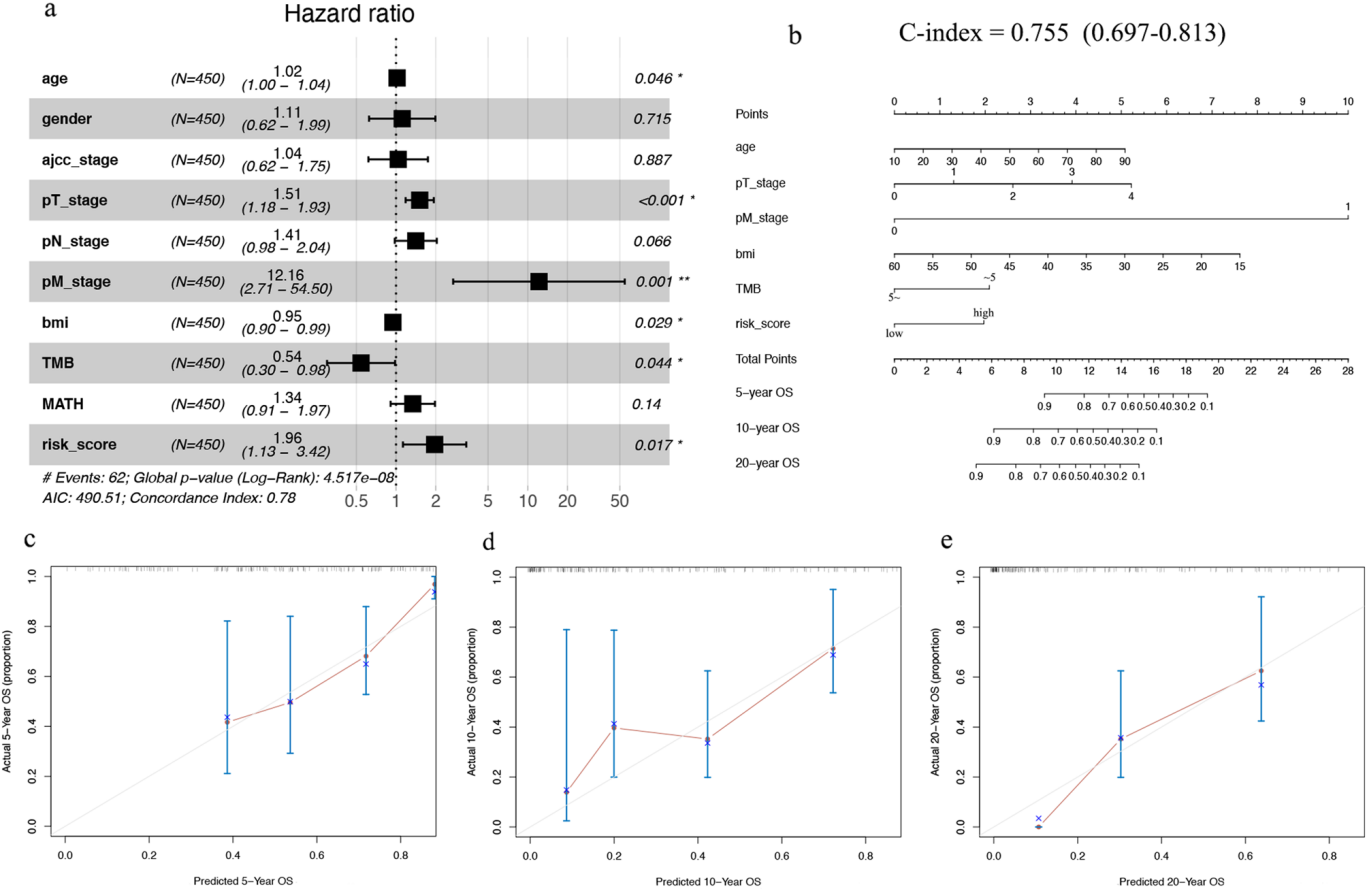
Predictive nomogram construction. (**a**) Hazard ratio and p‐value of the clinicopathological factors and risk scores involved in multivariate Cox regression in the overall cohort. (**b**) Nomogram to predict the overall survival rate of 5 years, 10 years and 20 years of patients with SKCM. (**c–e**) Calibration curve for the nomogram. *SKCM* skin cutaneous melanoma.

Considering the clinicopathological features and risk score, we developed a multivariate Cox regression risk forest plot for the overall cohort, which revealed that age, pT_stage, pM_stage, BMI, tumour mutation burden (TMB), and risk score were independent factors that affect the prognosis in patients with SKCM (Fig. 4a). A predictive nomogram predicted survival probability. This predicted OS well (C-index value: 0.755) at 5, 10, and even 20 years (Fig. 4b‒e).

### Functional enrichment analysis of ARG-based subtypes

**Figure 5:**
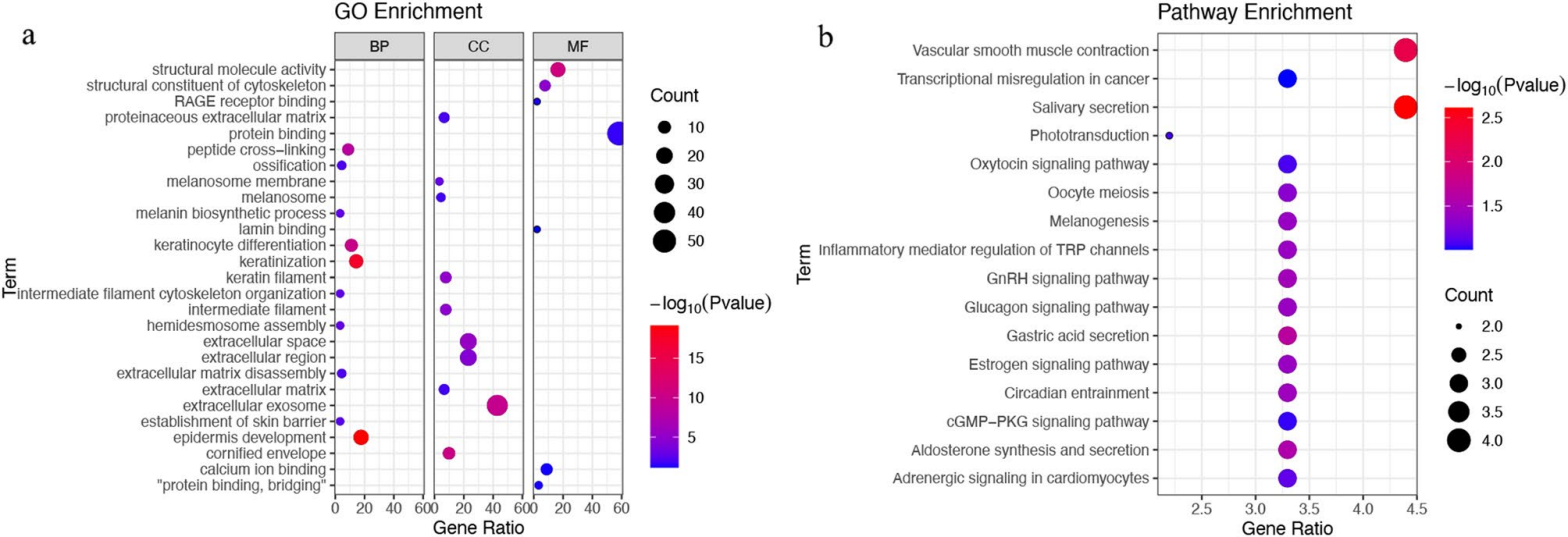
Functional enrichment analysis of DEGs associated with ageing in SKCM. (**a**) The enriched items in gene ontology analysis. (**b**) The enriched items in the Kyoto Encyclopedia of Genes and Genomes analysis. *DEG* differentially expressed gene, *SKCM* skin cutaneous melanoma.

We identified 92 age-related DEGs between the low/high-risk score groups (Fig. S2) to explore potential biological behaviours among different ageing patterns. The genes related to the cell ageing subtypes participated mainly in epidermis development, keratinisation, melanosome membrane formation, melanosome synthesis, structural molecule activity, and protein binding (Fig. 5a). Furthermore, the Kyoto Encyclopedia of Genes and Genomes (KEGG) pathway analysis revealed that these DEGs were mainly involved in salivary secretion, melanogenesis, regulation of inflammatory mediators of transient receptor potential channels, aldosterone synthesis and secretion, circadian entrainment, and vascular smooth muscle contraction (Fig. 5b).

### Relationships of risk score with somatic mutation and clinicopathological factors

**Figure 6:**
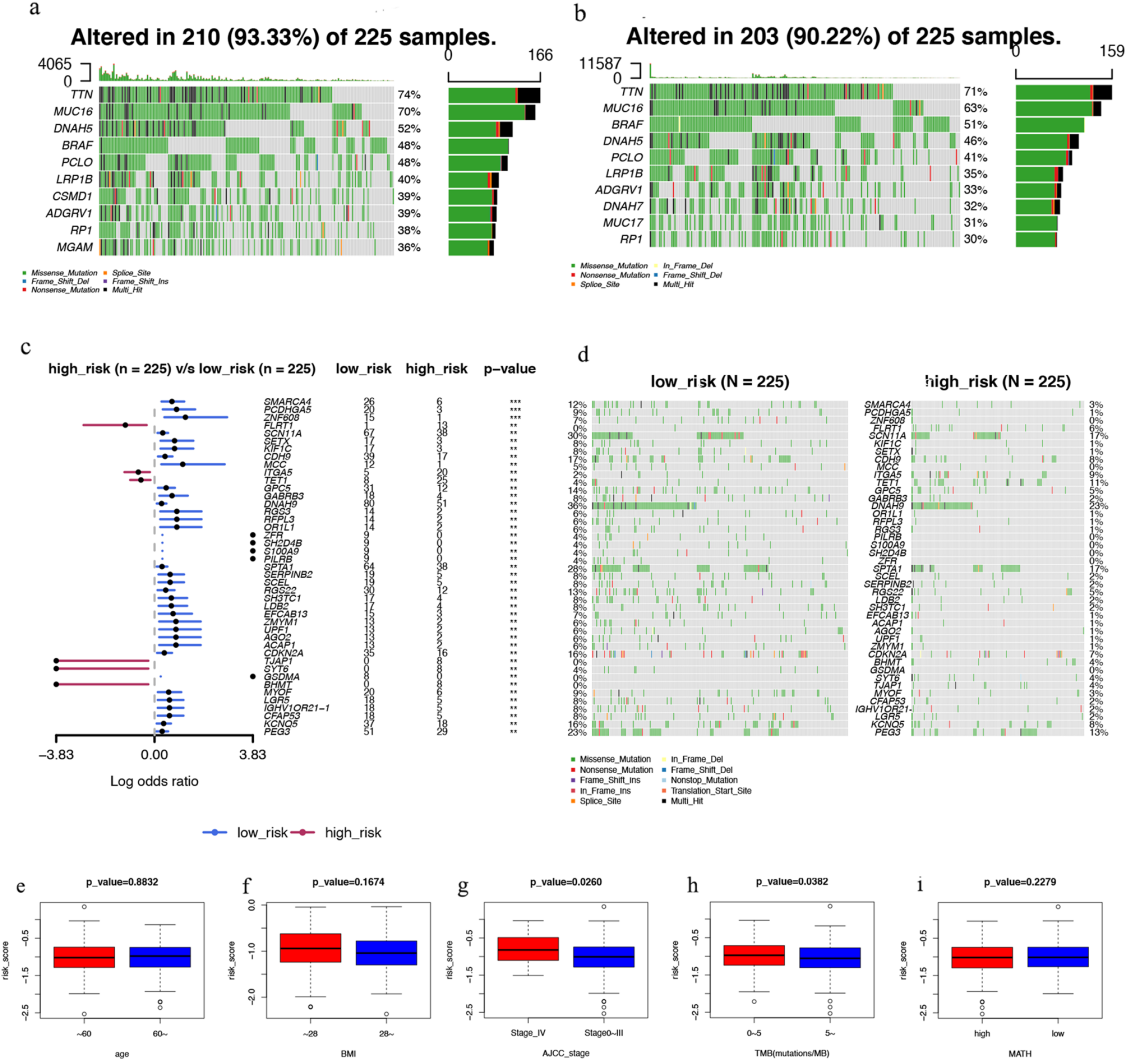
Association of the risk score with somatic mutations and clinicopathological factors. (**a–d**) Comparison of the frequency of somatic mutations in low- and high-risk groups. (**e–i**) Comparison of the risk score between the age, BMI, stage, TMB, and MATH subgroups. *BMI* body mass index, *TMB* tumour mutation burden, *MATH* mutant-allele tumour heterogeneity.

The distribution of somatic mutations between the two risk score subgroups was compared. *TTN*, *MUC16*, *DNAH5*, *BRAF*, and *PCLO* were the top five mutant genes in both the low- and high-risk groups (Fig. 6a,b). Mutations in *SMARCA4*, *PCDHGA5*, and *ZNF608* were more common in patients with a low-risk score than in those with a high-risk score. The opposite was observed for mutations in *FLRT1*, *ITGA5*, and *TET1* (Fig. 6c,d).

To investigate the influences of the risk score on clinical characteristics, we explored the relationship between clinical characteristics and the risk score (Fig. 6e‒i). The risk score was significantly higher in patients in the advanced stage (stage IV) than in patients in the early stages (stages 0‒III) (*p* = 0.0260). Patients with TMB ≤ 5 had a substantially higher risk score than those with TMB > 5 (*p* = 0.0382). The risk scores were similar among the subgroups according to age, BMI, and mutant-allele tumour heterogeneity (MATH).

### Correlation of risk scores with tumour immunity, tumour microenvironment, and drug sensitivity

**Figure 7:**
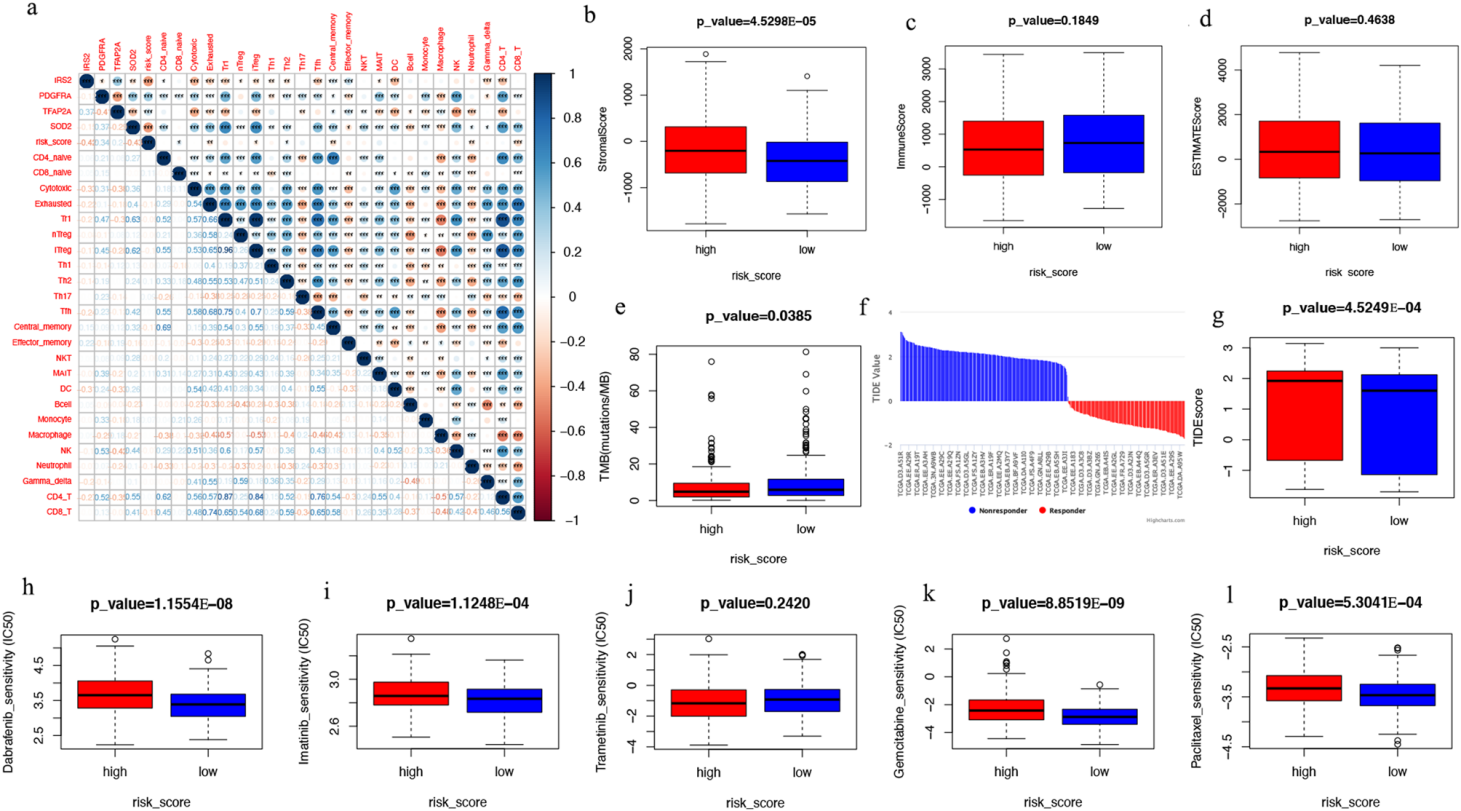
Tumours, immune microenvironment landscape, and drug efficacy prediction. (**a**) Correlation between the expression of ARGs and the degree of immune cell infiltration. (**b–d**) Association of risk score and tumour microenvironment. (**e–g**) Differences in TMB and TIDE scores between different risk score groups. (**h–l**) Sensitivity analysis of five common targeted and chemotherapeutic drugs between different risk score groups. *ARG* ageing-related gene, *TMB* tumour mutation burden, *TIDE* Tumour Immune Dysfunction and Exclusion.

We found a correlation between the expression of prognostic ARGs (*IRS2*, *PDGFRA*, *TFAP2A*, and *SOD2*) and immune infiltration in SKCM (Fig. 7a). *IRS2* expression was negatively correlated with the abundance of cytotoxic cells (r = − 0.33, *p* < 0.001) and dendritic cells (r = − 0.31, *p* < 0.001). *PDGFRA* expression was positively correlated with the abundance of NK (r = 0.53, *p* < 0.001) and CD4 T cells (r = 0.52, *p* < 0.001). *TFAP2A* expression was negatively correlated with the abundance of NK cells (r = − 0.42, *p* < 0.001) and cytotoxic cells (r = − 0.38, *p* < 0.001). Furthermore, the expression of *SOD2* was positively correlated with the abundance of Tr1 cells (r = 0.63, *p* < 0.001) and iTreg cells (r = 0.62, *p* < 0.001).

The matrix and immunological scores in the immune microenvironment were calculated using the ESTIMATE program. Although there were no significant differences in the ESTIMATE and immune scores between the two risk groups, the stromal score was considerably lower in the low-risk group than in the high-risk group (Fig. 7b‒d). The same trend of differences between groups was observed in the GSE65904 validation set, with the difference that the immune score was significantly lower in the high-risk score group compared to the low one (Fig. S3a‒c).

We then evaluated the response to immune checkpoint inhibitors in the high and low-risk score groups using the Tumour Immune Dysfunction and Exclusion (TIDE) tool. The TIDE score was significantly higher in patients with high-risk scores than in patients with low-risk scores (Fig. 7f,g). The same was true in the GSE65904 validation set (Fig. S3d,e).

Next, we evaluated the sensitivity of patients in the risk score subgroups to commonly used targeted and chemotherapy drugs. We found considerably lower semi-inhibitory concentration values of drugs other than trametinib (e.g. dabrafenib, imatinib, gemcitabine, and paclitaxel) administered to patients with low-risk score (Fig. 7h‒l). In patients with a low-risk score in the GSE65904 validation set, dabrafenib and gemcitabine had significantly lower semi-inhibitory concentration values (Fig. S3f–j). The results indicated that the risk score was correlated with the sensitivity of the drug.

### Construction of the lncRNA-miRNA-mRNA network

**Figure 8:**
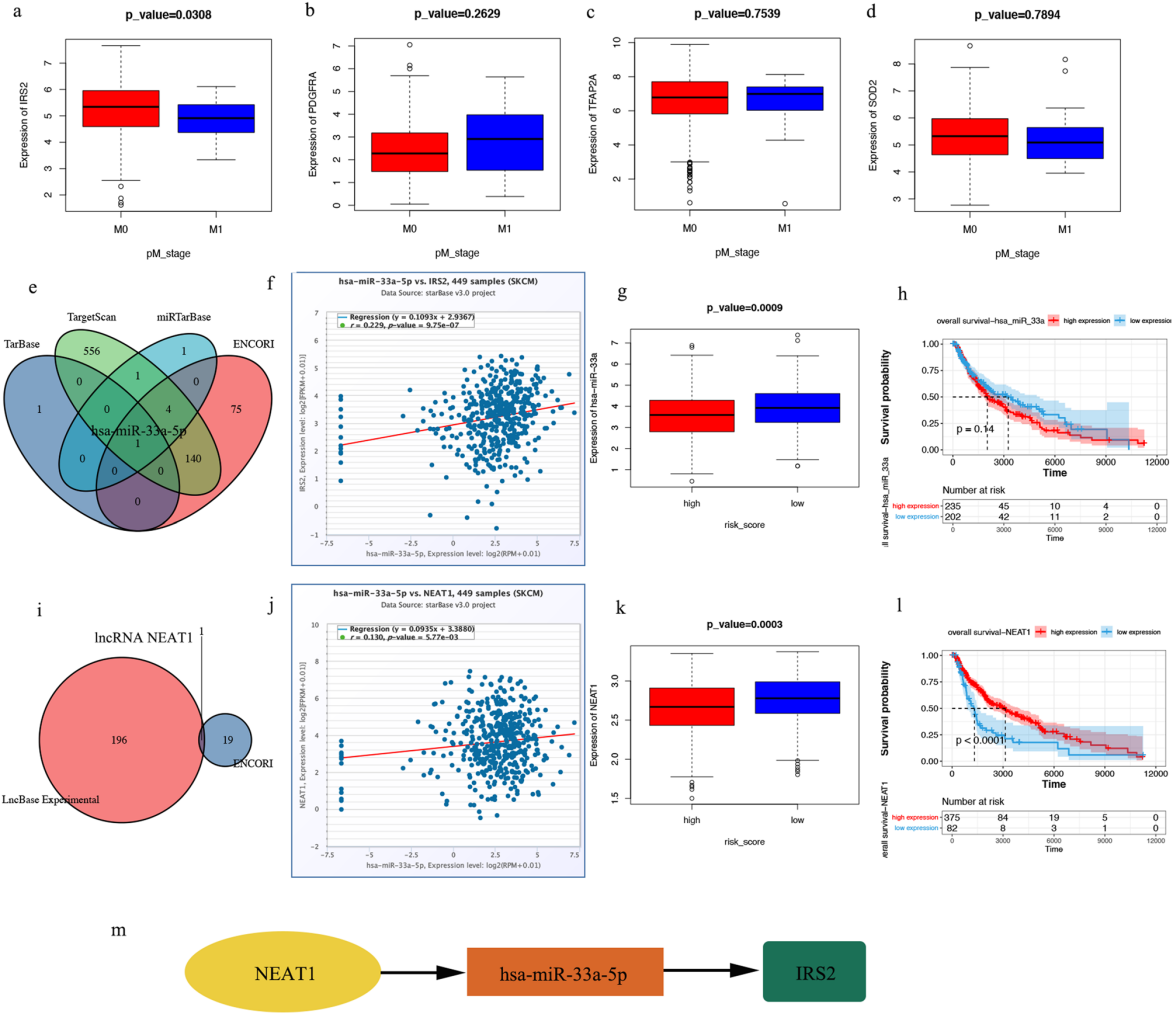
CeRNA network construction. (**a–d**) Comparison of four patients with ARGs expression levels between stage M0 and M1. (**e**) MiRNAs predicted by ENCORI, miRTarBase, TargetScan, and TarBase. (**f–h**) Has-miR-33a-5p expression level and prognostic value. (**i**) LncRNAs predicted by ENCORI and LncBase Experimental. (**j–l**) LncRNA *NEAT1* expression level and prognostic value. (**m**) The network of lncRNA-miRNA-mRNA. *ceRNA* competing endogenous RNA, *ARG* ageing-related gene, *miRNA* micro RNA, *lncRNA* long non-coding RNA.

As described above (Fig. 6g), risk score was closely correlated with distant metastasis of SKCM. Therefore, we further analysed the four ARGs that comprised the risk scores. *The expression level of IRS2* (Fig. 8a, p = 0.0308) expression level was correlated with distant metastasis. However, the expression of *PDGFRA* (*p* = 0.2629), *TFAP2A* (*p* = 0.7539), and *SOD2* (*p* = 0.7894) was not correlated with distant metastasis (Fig. 8b–d). Thus, *IRS2* may be involved in SKCM tumour metastasis.

To elucidate the potential molecular mechanisms of *IRS2* in SKCM, a network of lncRNA-miRNA-mRNA interaction was constructed. We identified miR-33a-5p as a targeted miRNA that binds to *IRS2* based on the data from ENCORI, miRTarBase, TargetScan, and TarBase (Fig. 8e, Table S3). *IRS2* expression was positively correlated with miR-33a-5p expression levels in the TCGA SKCM cohort (Fig. 8f, p = 9.75e−07). miR-33a-5p was down-regulated in the high-risk score group (Fig. 8g, p = 0.0009), but its expression did not correlate with OS (Fig. 8h, p = 0.14).

We constructed a lncRNA-miRNA-mRNA axis based on the above result and its upstream lncRNA targets (Table S4). The lncRNA *NEAT1* was defined as a target, as shown in Fig. 8i, and miR-33a-5p was positively correlated with *NEAT1* expression levels (Fig. 8j, p = 5.77e−03). *NEAT1* was significantly down-regulated in the high-risk score group (Fig. 8k, p = 0.0003), and patients with SKCM with high levels of *NEAT1* had significantly better OS (Fig. 8l, p < 0.0001). Figure 8m demonstrates the ceRNA network. The regulatory axis of the lncRNA *NEAT1*/miR-33a-5p/*IRS2* may be crucial to the evolution of SKCM.

## Discussion

Given the bidirectional role of cell ageing in tumours, ARGs may be a potential prognostic and therapeutic target in cancer^8, 15–17^ as in hepatocellular carcinoma^18^. We investigated the role of ARGs in SKCM since this was previously unclear. First, we elucidated the expression and mutation of ARGs in SKCM. We identified 87 differentially expressed ARGs. We constructed an ARG prognostic risk-scoring model based on RNA-seq, mutation, and clinical data from patients with SKCM in TCGA, which included *IRS2*, *PDGFRA*, *TFAP2A*, and *SOD2*; we distinguished patients with low and high-risk scores accurately based on these criteria. Patients with a low-risk score could benefit more from immunotherapy and commonly used targeted and chemotherapy drugs. Immune cell infiltration and tumour microenvironment differed substantially between the high- and low-risk score groups. Age, pT_stage, pM_stage, BMI, TMB, and our ARG-based risk score were independent factors affecting the prognosis of patients with SKCM. These factors were included in a prognosis nomogram. We also constructed a lncRNA *NEAT1*/miR-33a-5p/*IRS2* regulatory axis of the ceRNA network to explore the mechanism of metastasis progression of SKCM.

Regarding mutation of ARGs, we found that the mutation frequency of *LRP2* in all patients reached 22%. Interestingly, there was a relatively consistent synergistic effect among the 87 ARG mutations, indicating that ageing is a biological process regulated by multiple genes. When more ARG mutations accumulated, the SKCM occurrence became more likely. *NRAS, BRAF, IDH1, MAP2K1, RQCD1, HBD, KRAS, RAC1,* and *SPANXB1* were identified as potential driver genes in SKCM.

Our prognostic risk-scoring model, including *IRS2, PDGFRA*, *TFAP2A*, and *SOD,* was constructed to accurately predict the OS of patients with SKCM. In 2006, Winnepenninckx et al.^19^ conducted a microarray analysis of the gene expression profile in patients with melanoma and demonstrated that the expression of *MCM4* and *MCM6* was closely correlated with total survival. With advances in sequencing technology, prognostic markers were investigated^20–22^. For example, Yang et al. developed and verified glycolysis and immune-related prognostic features in SKCM in 2021. Another study showed that genes related to pyroptosis could help diagnose and predict survival in patients with SKCM^23^. Furthermore, the prognostic features associated with ferroptosis can predict the prognosis in patients with SKCM^24^. In this study, we characterised age-related prognostic gene features of SKCM, which were validated in both internal and external sets; this has not been previously reported. These findings provide additional options for prognosis prediction in SKCM. Furthermore, we found that age, pT_stage, pM_stage, BMI, and TMB correlated with the prognosis in patients with SKCM. We designed a predictive nomogram based on these factors that predicted the OS rate at 5, 10, and even 20 years.

To investigate the role of prognostic ARGs in SKCM, we performed a functional enrichment analysis on DEGs between the low- and high-risk score groups. These ARGs were mainly involved in melanin biosynthetic processes (e.g. melanosome synthesis and melanosome membranes) and transcriptional misregulation in cancer, suggesting that cell ageing may play a crucial role in the evolution of SKCM.

Another important finding of our study is that the four prognostic ARGs in the model were significantly correlated with the degree of infiltration of tumour immune cells. The relationship between risk score and tumour microenvironment further confirmed the role of ageing in the tumour microenvironment. Gravekamp et al.^25^ reported that age-related changes in the immune system are caused by slow T cell responses, whereas decreased immunity in older individuals is caused by the lack of naïve T cells and impaired activation of T cell and antigen-presenting cell pathways. Immunotherapy is becoming more popular as a cancer treatment strategy. TMB levels and immune checkpoint levels (e.g., CTLA4, PD-L1, PD-1) are used as biomarkers to predict the efficacy of immunotherapy (Fig. 7e–g). Patients with high TMB will benefit from immunotherapy due to the increased number of new antigens. The role of TMB has been demonstrated in PD-1/PD-L1 antibody therapy for malignant tumours with mismatch repair defects^26, 27^. TIDE, a novel algorithm, can predict the efficacy of immune checkpoint inhibitors better than a single marker by combining two immune escape mechanisms of tumours (rejection and immune dysfunction)^28^. With this in mind, we evaluated the efficacy of immunotherapy in patients with SKCM, suggesting that patients with low-risk scores might benefit more from immunotherapy.

Although immunotherapy can help numerous people with SKCM, some patients with SKCM still do not get ideal results despite taking immune checkpoint inhibitors^29, 30^. To improve their prognosis, patients with SKCM should return to traditional molecular targeted therapy and chemotherapy due to the existing treatment bottleneck. Therefore, the mRNA expression data of the TCGA and Gene Expression Omnibus (GEO) database were used to investigate and evaluate the sensitivity of the risk score to conventional drugs (dabrafenib, imatinib, trametinib, gemcitabine, and paclitaxel) used in patients with SKCM. We showed that patients in the low-risk score group may fare better than those in the high-risk score group with the same medications. Currently, targeted therapy combined with chemotherapy is an option for advanced treatment of SKCM. However, the existence of heterogeneity and resistance mechanisms makes drug treatment less effective than it should be, and our results offer a new scope for research and development of highly effective drugs.

In analysing the relationship between the risk score and clinical factors, contrary to our expectation, there was no correlation between the risk score and the age of the patient in this study (Fig. 6e), which may be related to the original intention of the risk score system we established, we built the risk score prediction model based on the OS of the patients and did not consider age, so the ARGs screened did not necessarily correlate strongly with age. In addition, we calculated the correlation between the expression levels of each of the four ARGs and the age factor, and the results suggested that the correlation coefficients of IRS2, PDGFRA, TFAP2A, and SOD2 with age were 0.01, − 0.18, 0.2, and − 0.08, respectively (Fig. S4). This suggests that at least the expression levels of PDGFRA and TFAP2A are correlated with age, but weighted calculations to obtain risk scores may cancel out the correlation with age between each other. And when analysing the relationship between the risk score and the clinical staging, we observed that the risk score of advanced-stage patients was considerably higher than early-stage patients, suggesting that risk scores might be closely correlated with distant SKCM metastases. Among the four ARGs that comprise the risk score, *IRS2* had low expression in patients with SKCM with distant metastases. To explore the mechanism of *ISR2* in regulating SKCM metastasis, we also constructed a lncRNA-miRNA-mRNA network and confirmed a regulatory axis of the lncRNA NEAT1/miR-33a-5p/IRS2 after strict screening. A previous study showed that miR-33a-5p could inhibit the growth, metastasis, and EMT of SKCM cells and the activation of the PI3K/AKT/mTOR pathway by targeting *SNAI2*^31^. Interestingly, the NEAT1 lncRNA can promote the proliferation, invasion, and migration of SKCM cells by regulating miR-495-3p and *E2F3*^32^. In our study, *NEAT1* expression was also found to be correlated with the prognosis of patients with SKCM. Overall, evidence suggests that the regulatory axis of the lncRNA NEAT1/miR-33a-5p/IRIS2 may play an important role in the metastasis of SKCM.

This study had several limitations. First, the current risk score was established and validated based on public databases and should be validated using prospectively collected data. Second, considering that SKCM is a typical polygenic disease, there is an intrinsic bias when using only ARGs to establish the prognostic model. Finally, the regulatory relationship of ceRNA needs to be confirmed by in vivo and in vitro experiments, as is currently being done.

In conclusion, we conducted a comprehensive and systematic bioinformatics analysis and established a prognostic scoring model containing four ARGs (*IRS2*, *PDGFRA*, *TFAP2A,* and *SOD2*). Grouping based on the scoring system predicts the prognosis of patients with SKCM and provides information on patients' sensitivity to immunotherapy, targeted therapy, and chemotherapy. This can assist in the formulation of individualised treatment strategies and provide new avenues for the research and development of new drugs. Our findings also revealed a regulatory axis involving the lncRNA *NEAT1*/miR-33a-5p/*IRS2*, which may be crucial in SKCM metastasis. This result must be confirmed through further research.

## Materials and methods

### Data collection and analysis

The graphical abstract for this study is shown in Fig. S5. First, 307 human ageing-related genes were downloaded from Human Ageing Genomic Resources^33^ (Table S1). The TCGA database was then used to obtain RNA-seq data, micro RNA (miRNA), somatic mutation, and clinical prognosis data for 471 patients with SKCM with the R package ‘TCGAbiolinks’^34^, whereas the Genotype-Tissue Expression (GTEx) database was used to retrieve RNA-seq data from 1305 normal skin samples. TCGA and GTEx RNA-seq data were combined to eliminate batch effect and were standardised with the R package ‘limma’ [17] (Fig. S6). Raw count from RNA-seq data were converted to TPM format for inter-group comparison of gene expression levels. The GSE65904 dataset from the GEO database was used as an external validation set.

### Differential expression identification and functional enrichment analysis of ARG

Differentially expressed genes (DEGs) in SKCM and normal skin tissues were identified using the ‘limma’ package^35^. Their intersection with previously obtained ARGs was obtained to identify differentially expressed ARGs (Table S1). Then, we built a protein–protein interaction (PPI) network for the ARGs obtained using STRING and calculated the hub genes using Cytoscape (https://cytoscape.org/). The functional enrichment analysis of DEGs was performed using DAVID and visualised using the R package ‘ggplot2’^36^ to further examine the probable function of DEGs related to the patterns of cellular ageing and to find relevant gene functions and enrichment pathways.

### Establishment and validation of an ARG-based prognostic model

First, genes with a strong predictive value in expression were chosen from differentially expressed ARGs for further analysis using the LASSO regression analysis in the R package ‘glmnet’^37^. In the LASSO model, the expression levels of differentially expressed ARGs in the training set were used as the independent variable (x) and the OS and survival status of the patients in the training set were used as dependent variables (y). Multivariate Cox regression analysis was used to further restrict the potential gene range based on these predictive ARGs in the R package ‘survival’^38^. Risk score = Σi(coef(i) × exp(i)), where coef is the coefficient and exp is the expression level of each candidate gene.

The TCGA SKCM data were randomly split into a training and internal validation set at a 2:1 ratio. Clinicopathological characteristics were not biased between the two sets (Table S5). The training set was used to calculate the risk scores. Based on the median risk score, patients with SKCM were divided into low-risk and high-risk subgroups, and OS between the two categories was compared using Kaplan–Meier analysis. The receiver operating characteristic (ROC) curve analysis was used to assess the accuracy of the prediction of the risk score. For the examination of ROC curves at 5, 10, and 20 years, the R package ‘survivalROC’^39^ was used.

Validation was performed in the validation set, overall queue, and the GSE65904 dataset. A risk regression forest map was plotted based on clinical features, risk score, and mutation data. Then a prognosis nomogram was plotted for patients with SKCM using the R package ‘rms’ (https://CRAN.R-project.org/package=rms).

### Clinical correlation of prognostic risk score

The relationships between clinical features (age, body mass index [BMI], TNM stage, TMB, and MATH^40, 41^) and risk score were investigated using two-tailed t-tests. To evaluate whether the risk score was independent of other clinicopathological variables, we used a multivariate Cox regression analysis.

### Analysis of immune microenvironment, mutation status, tumour heterogeneity, and drug sensitivity

For the immune-related analysis, we determined the infiltration scores of 24 immune cell types using the ImmuneCellAI database based on TCGA RNA-seq data and visualised the correlations of the risk score and candidate ARGs with the degree of immune cell infiltration using the R package ‘corrplot’(https://CRAN.R-project.org/package=corrplot). The composition of tumour stromal cells and immune cells was assessed using the R package ‘estimate’^42^.

A waterfall graph of the frequency of ARGs mutations was generated in patients with SKCM using the R package ‘maftools’^43^, which compared the mutation status of ARGs between the low-risk and high-risk subgroups, explored the synergy and mutual exclusion of driver genes and mutations in ARG in patients with SKCM, and then calculated the TMB and MATH of each patient in the two groups.

To explore the difference in the therapeutic effects of drugs on the two groups of patients, we used the online tool TIDE [26] to estimate patients’ responses to anti-PD1 and anti-CTLA4 immunotherapy. We used the ‘pRRophetic’^44^ R package to calculate the IC50 values of the chemotherapy and the targeted drugs commonly used for malignant melanoma.

### Construction of a network of competing endogenous RNAs

A competitive endogenous RNA (ceRNA) network was built to determine the role ARGs might have in SKCM. Databases such as ENCORI, miRTarBase, TargetScan, and TarBase were used to predict miRNA targets that bound to ARGs. Long non-coding RNA (lncRNA) targets interacting with miRNAs were predicted based on the ENCORI and LncBase databases. Finally, the TCGA dataset was used to investigate the expression and prognostic value of potential miRNAs and lncRNAs.

### Statistical analysis

R version 4.0.3 for Mac was used to perform all statistical analyses. The threshold for statistical significance was fixed at *p* < 0.05.

## Supplementary Information

Supplementary Figure S1.

Supplementary Figure S2.

Supplementary Figure S3.

Supplementary Figure S4.

Supplementary Figure S5.

Supplementary Figure S6.

Supplementary Table S1.

Supplementary Table S2.

Supplementary Table S3.

Supplementary Table S4.

Supplementary Table S5.

Supplementary Legends.

## Data Availability

The datasets analysed during the current study are available in the Cancer Genome Atlas (https://portal.gdc.cancer.Gov/), the Genotype-Tissue Expression project (https://www.gtexportal.org/home/), and the Gene Expression Omnibus (https://www.ncbi.nlm.nih.gov/geo/). Furthermore, the codes used in the study can be viewed in Mendeley Data (http://dx.doi.org/10.17632/zcmr4jkzwr.1). All data supporting the findings of the study are available from the corresponding author on a reasonable request.
