# Supplementary figures and images for: Identification of the ageing‐related prognostic gene signature, and the associated regulation axis in skin cutaneous melanoma
Source: Sci Rep. 2023 Jan 11;13:24. doi: 10.1038/s41598-022-22259-0 (PMC9834281; doi:10.1038/s41598-022-22259-0)

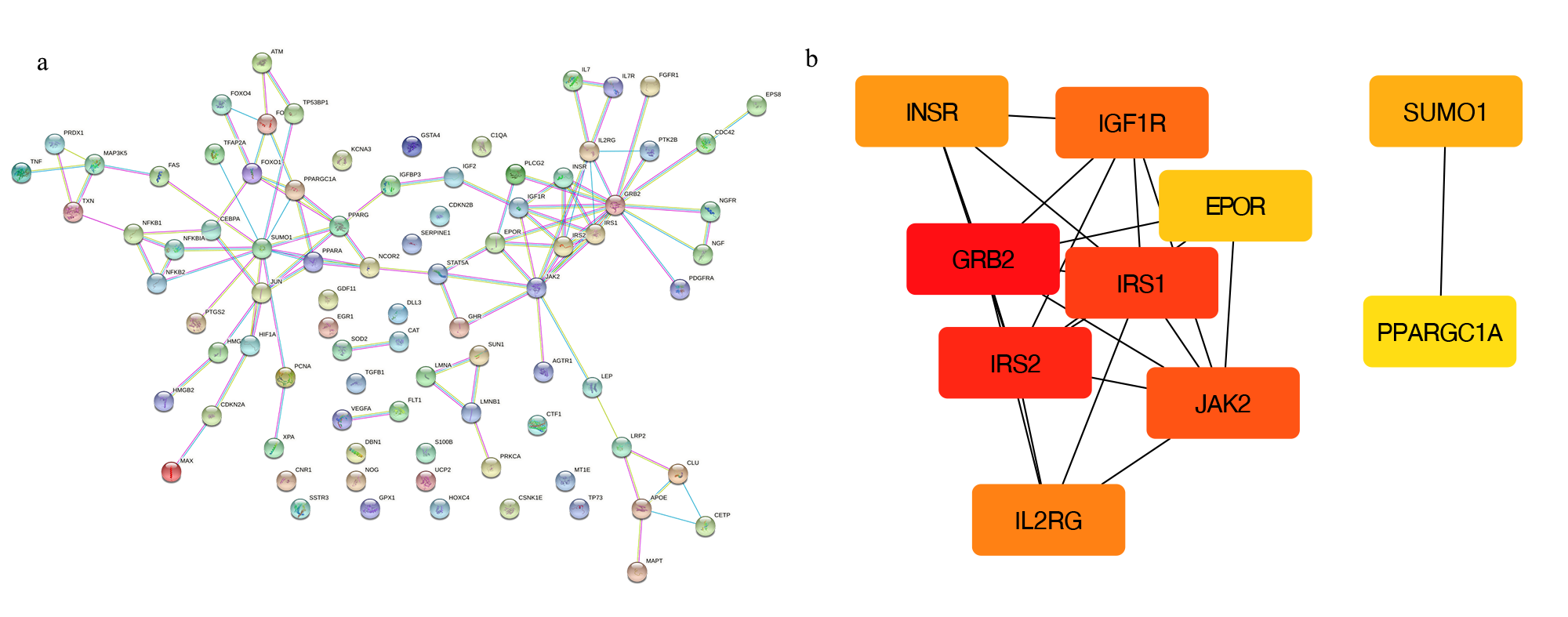

Supplement: Supplementary file 1 — Supplementary Figure S1. [file 41598_2022_22259_MOESM1_ESM.tif]

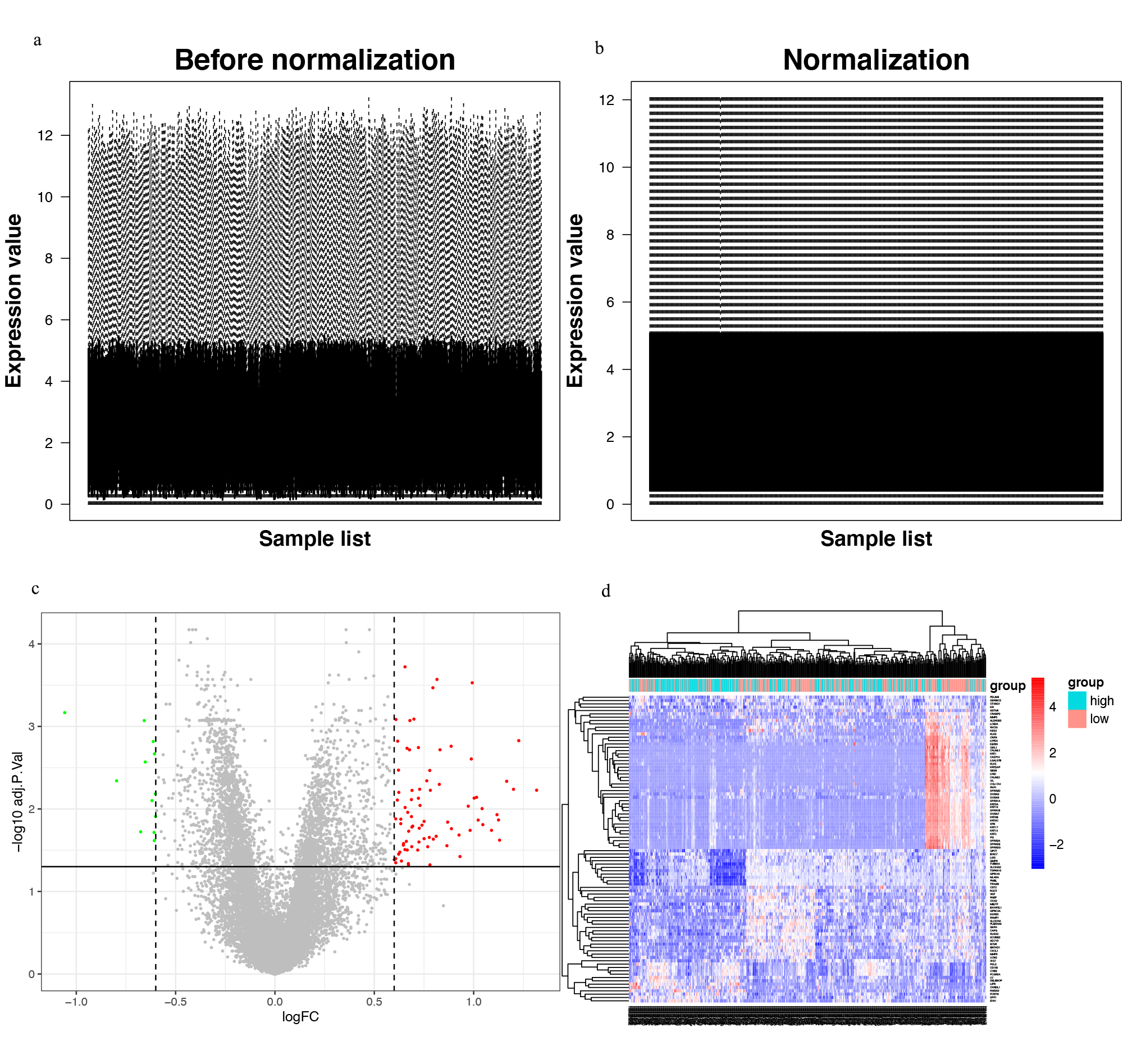

Supplement: Supplementary file 2 — Supplementary Figure S2. [file 41598_2022_22259_MOESM2_ESM.tif]

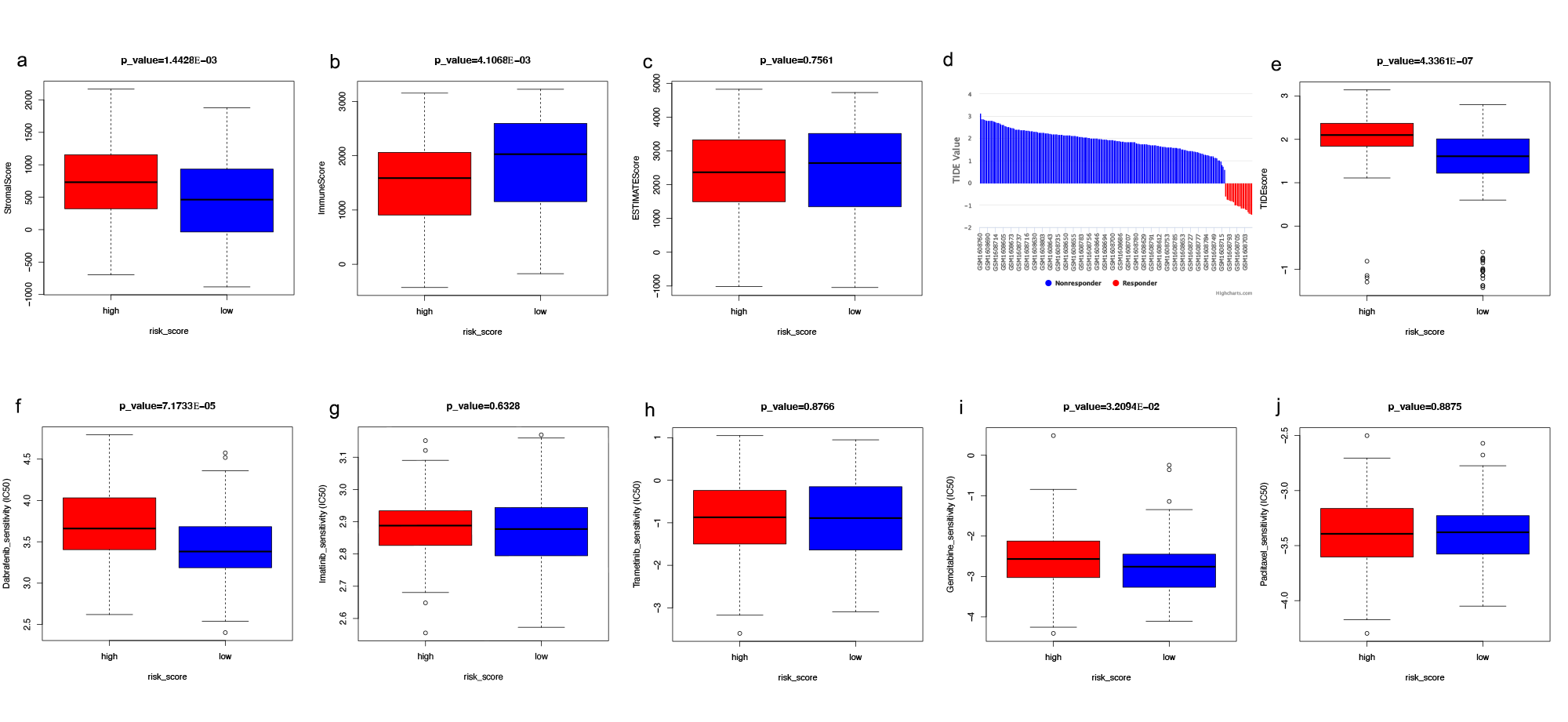

Supplement: Supplementary file 3 — Supplementary Figure S3. [file 41598_2022_22259_MOESM3_ESM.tif]

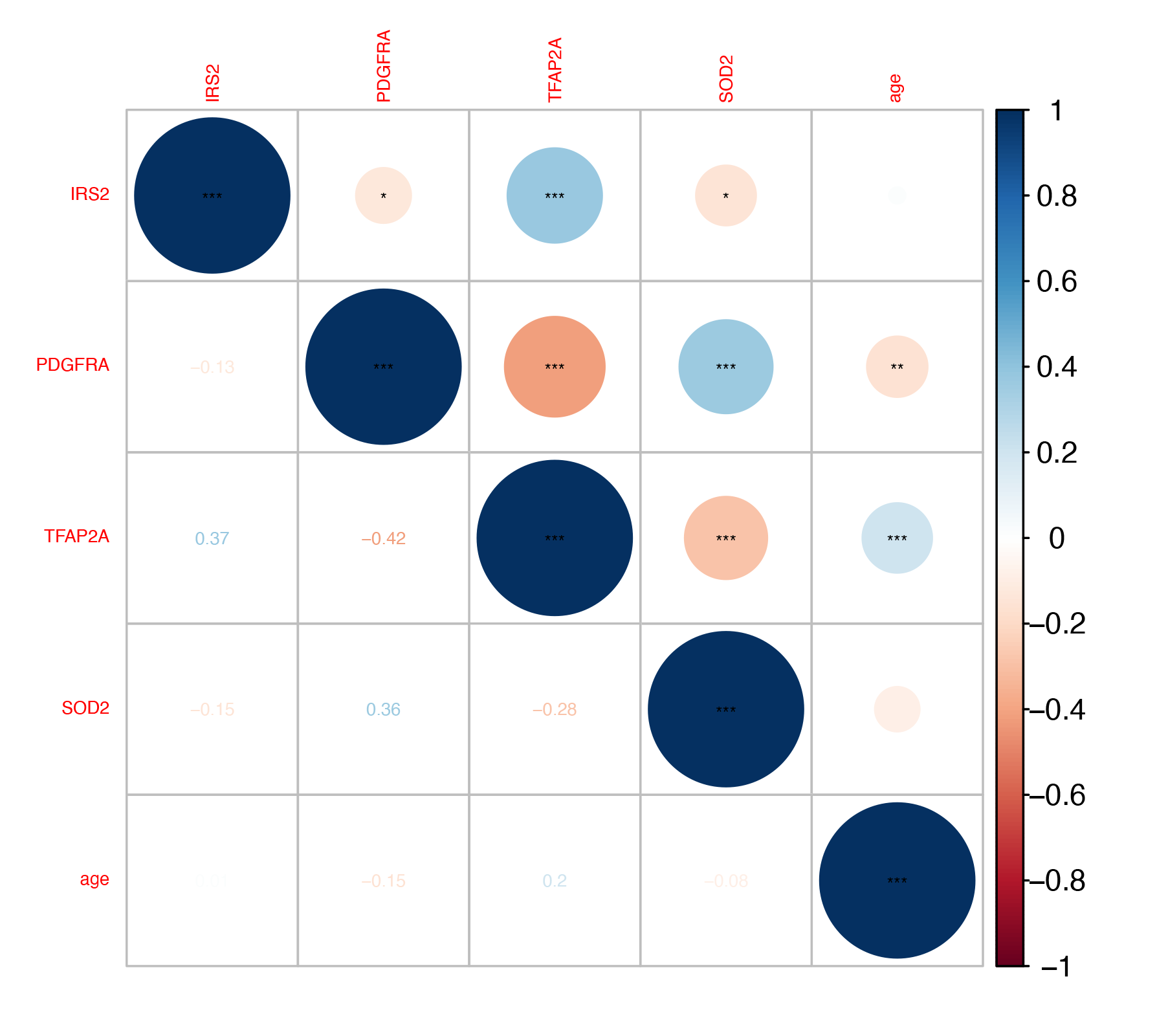

Supplement: Supplementary file 4 — Supplementary Figure S4. [file 41598_2022_22259_MOESM4_ESM.tif]

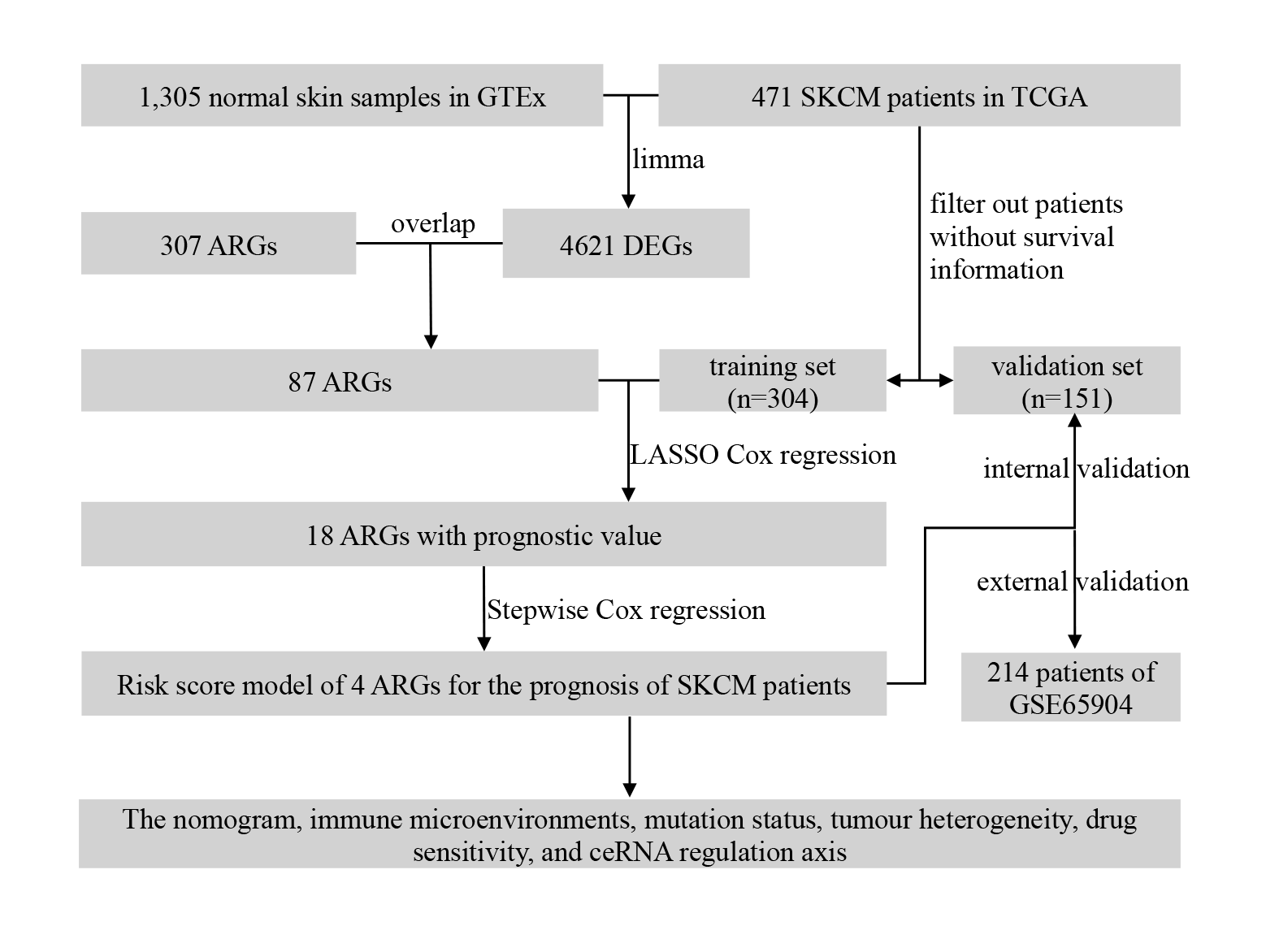

Supplement: Supplementary file 5 — Supplementary Figure S5. [file 41598_2022_22259_MOESM5_ESM.tif]

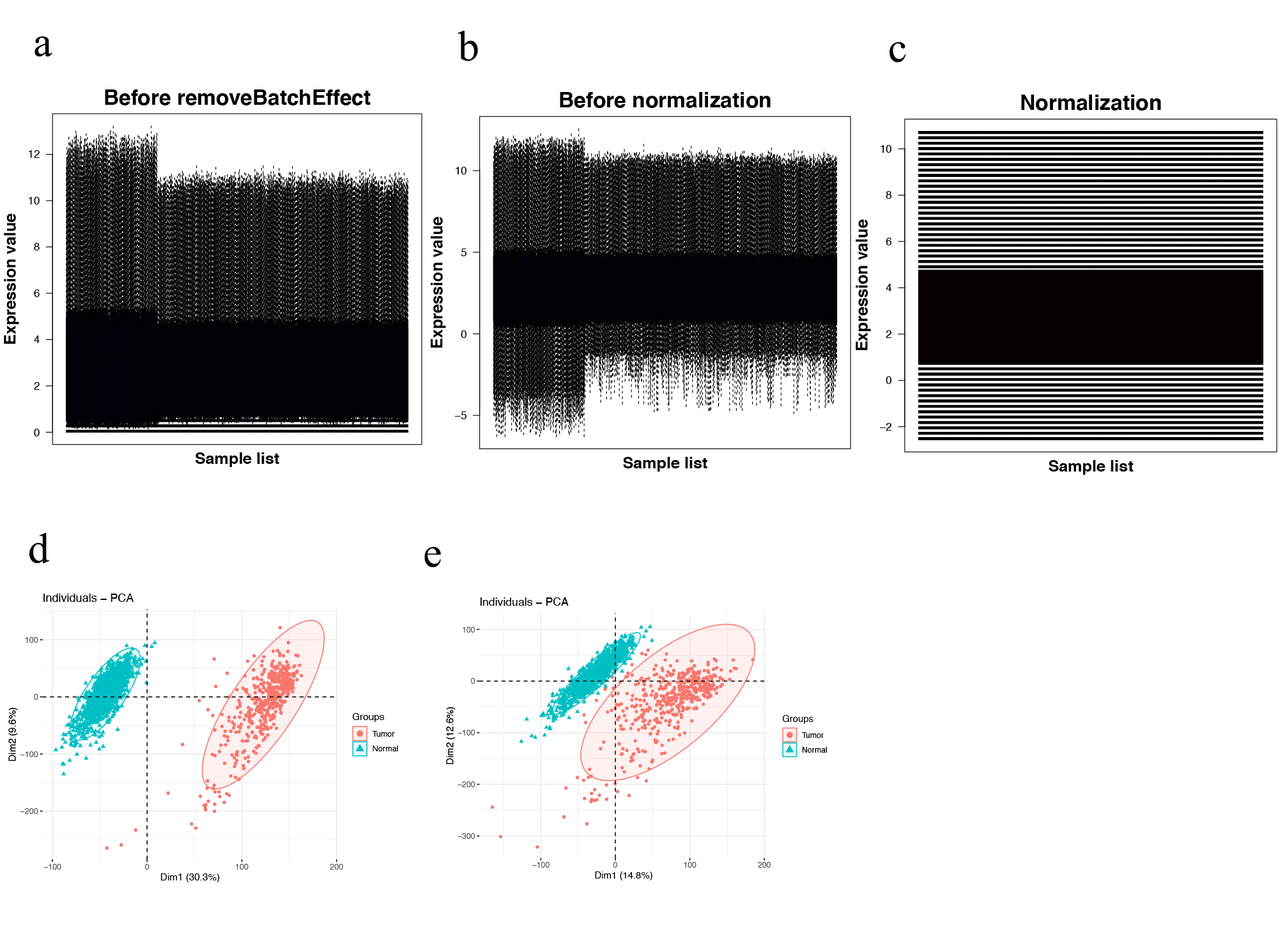

Supplement: Supplementary file 6 — Supplementary Figure S6. [file 41598_2022_22259_MOESM6_ESM.tif]
