# Supplementary material for: Identification of the ageing‐related prognostic gene signature, and the associated regulation axis in skin cutaneous melanoma
Source: Sci Rep. 2023 Jan 11;13:24. doi: 10.1038/s41598-022-22259-0 (PMC9834281; doi:10.1038/s41598-022-22259-0)
Supplement: Supplementary file 11 — Supplementary Table S5. [file 41598_2022_22259_MOESM11_ESM.docx]

Table S5. Comparison of clinicopathological characteristics of patients in the training set and the Validation set

| Characteristics | Training set (n=304) | Validation set (n=151) | P value (Chi-Square Test) |
| --- | --- | --- | --- |
| Age (years) |  |  | 0.694 |
| Median (Range) | 58（15 - 87） | 59（23 - 87） |  |
| ≤ 60 | 169 | 81 |  |
| > 60 | 135 | 70 |  |
| Gender |  |  | 0.660 |
| Male | 191 | 42 |  |
| Female | 113 | 28 |  |
| BMI |  |  | 0.425 |
| Median (Range) | 27.34（17.78 - 55.47） | 26.58（18.42 – 46.04） |  |
| ≤ 28 | 88 | 51 |  |
| > 28 | 70 | 28 |  |
| Unknown | 146 | 72 |  |
| AJCC stage |  |  | 0.471 |
| Stage 0 | 4 | 2 |  |
| Stage Ⅰ | 57 | 19 |  |
| Stage Ⅱ | 85 | 51 |  |
| Stage Ⅲ | 112 | 58 |  |
| Stage Ⅳ | 14 | 9 |  |
| Unknown | 32 | 12 |  |
| Race |  |  | 0.427 |
| Asian | 10 | 2 |  |
| White | 288 | 145 |  |
| Unknown | 6 | 4 |  |
| Overall survival (days) |  |  | 0.843 |
| Median (Range) | 1160（6 - 11217） | 1096（7 - 11252） |  |
| ≤ 1095 days (3 years) | 148 | 75 |  |
| > 1095 days (3 years) | 156 | 76 |  |
