# Supplementary material for: Identification of the ageing‐related prognostic gene signature, and the associated regulation axis in skin cutaneous melanoma
Source: Sci Rep. 2023 Jan 11;13:24. doi: 10.1038/s41598-022-22259-0 (PMC9834281; doi:10.1038/s41598-022-22259-0)
Supplement: Supplementary file 12 — Supplementary Legends. [file 41598_2022_22259_MOESM12_ESM.docx]

**Figure S1. Mapping protein-protein interaction networks and calculation of hub genes.**

**Figure S2. Normalisation of expression data between groups with high and low-risk score, and volcano and heat maps of DEGs between groups.**

DEG, differentially expressed gene

**Figure S3. Immune microenvironment landscape and drug efficacy prediction in GSE65904. a–c** Association of risk score and tumour microenvironment. **d, e** Differences in TIDE scores between different risk score groups. **f–j** Sensitivity analysis of five common targeted and chemotherapeutic drugs between different risk score groups.

TIDE, Tumour Immune Dysfunction and Exclusion

**Figure S4. Correlation of the four prognostic ARGs with age.**

**Figure S5.** **The overall design and workflow of the study.**

**Figure S6. TCGA and GTEx data merge processing. a–c** Removal of batch effects and normalisation. **d, e** Principal component analysis before and after removal of batch effects.

TCGA, The Cancer Genome Atlas
